# The Masquerading Myocarditis: A Case of Late Recurrence of Acute Myocarditis Presenting as “Peripartum Cardiomyopathy”

**DOI:** 10.7759/cureus.57782

**Published:** 2024-04-07

**Authors:** Saint-Martin Allihien, Sammudeen Ibrahim, Walter Y Agyeman, Favour Markson, Amal Naji, Catherine N Marti

**Affiliations:** 1 Internal Medicine, Piedmont Athens Regional Medical Center, Athens, USA; 2 Medicine, Piedmont Athens Regional Medical Center, Athens, USA; 3 Internal Medicine, Lincoln Medical Center, Bronx, USA; 4 Heart Failure and Transplantation Cardiology, Piedmont Heart Institute Athens, Athens, USA

**Keywords:** autoantibodies, endomyocardial biopsy, peripartum cardiomyopathy, myocarditis, chest pain

## Abstract

Myocarditis is a potentially fatal medical condition with varied etiologies. Peripartum cardiomyopathy (PPCM) refers to systolic dysfunction occurring toward the end of pregnancy or in the months following delivery; it is a diagnosis of exclusion. We present a patient with chest pain, bipedal edema, markedly elevated troponins, electrocardiogram (EKG) findings that were concerning for myocardial infarction, and a significantly reduced left ventricular ejection fraction (LVEF) on the echocardiogram. The patient's presentation in the postpartum period closely resembled peripartum cardiomyopathy and presented a peculiar diagnostic challenge to our team. The right diagnosis was possible with cardiac magnetic resonance imaging, which revealed late gadolinium enhancement. Additionally, the patient had positive Coxsackie B5 and Epstein Bar virus serologies. While the clinical course of the disease is often benign, it could rapidly deteriorate, so early recognition and diagnosis are important to ensure patients receive adequate therapeutic support.

## Introduction

Myocarditis refers to inflammation of the myocardium. Possible etiologies include infection, immune system activation, or medication adverse effects. Clinically, we classify myocarditis as acute when symptoms manifest within a month; beyond this time frame, we classify it as chronic inflammatory myocarditis [1]. Viral etiologies are the leading cause of this rare disease, with the pre-COVID incidence reported as 1 to 10 cases per 100,000. COVID-19 disease is believed to have caused an exponential increase in the incidence of the disease [2]. The disease has a low recurrence rate, with most recurrences occurring within a year of the initial diagnosis, while late recurrence is rare [3].

In contrast to myocarditis, peripartum cardiomyopathy (PPCM) is a form of systolic heart failure with reduced ejection fraction that affects women toward the end of pregnancy or in the months following delivery, where no other cause of heart failure is found. While viral myocarditis has been suggested as a possible cause of peripartum cardiomyopathy, the etiology of PPCM remains poorly understood with no proven mechanism, and both myocarditis and PPCM are recognized as distinct pathologies [4].

Herewith, we present a rare case of late recurrence of myocarditis presenting as "peripartum cardiomyopathy" five years after the initial diagnosis. The diagnostic challenge of properly classifying the patient’s presentation for appropriate treatment makes this case report one of significant learning value.

## Case presentation

A 26-year-old female with a history of asthma was diagnosed with infective myocarditis five years prior to the current presentation (we were unable to obtain records about the specific infection). Cardiovascular magnetic resonance imaging (CMRI) confirmed the diagnosis at that time, revealing scattered late gadolinium enhancement consistent with myocarditis. The ejection fraction was 57% at the time of diagnosis, and the coronary angiogram showed no evidence of coronary artery disease. She had been well thereafter, and a follow-up MRI 16 months later showed improved late gadolinium enhancement.

**Figure 1:**
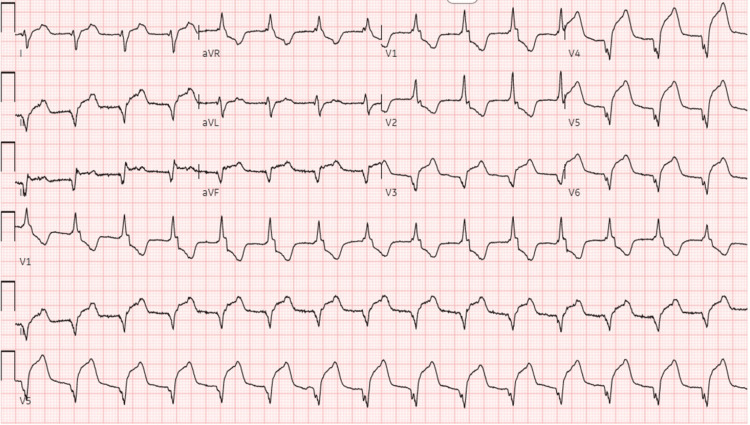
Electrocardiogram showing accelerated junctional rhythm and anterolateral infarct.

Twelve days prior to her index presentation, she had a premature delivery at 28 weeks gestation on account of severe preeclampsia and HELLP (hemolysis-elevated liver enzymes and low platelet) syndrome. On presentation, the patient had remained on Nifedipine 30 mg, which was prescribed for preeclampsia. She reported to the emergency department with chest pain. She woke up that morning with moderate, non-radiating chest pain, alleviated by sitting up and, worse, by lying on her back. The physical examination was remarkable for mild bilateral pitting edema. Her breathing was not labored; breath sounds were vesicular, with adequate air entry bilaterally. The first and second heart sounds were present, with no added heart sounds or murmurs. The rest of the physical exam was unremarkable. An electrocardiogram (EKG) revealed an accelerated junctional rhythm, antero-lateral ST elevations, and prolonged QT (Figure 1).

**Figure 2:**
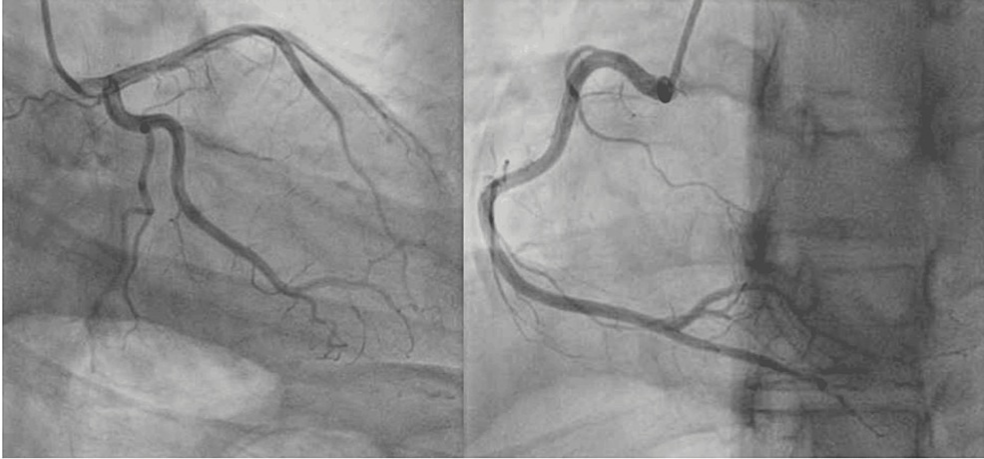
Coronary angiogram still frames with no evidence of artery stenosis.

The patient's troponins markedly elevated at 23,949 ng/L and trended upward, while her B-type natriuretic peptide (BNP) remained within the normal limit (Table 1). While the patient had no risk factors for coronary artery disease, there was a concern for possible spontaneous coronary dissection given her postpartum status and recent elevated blood pressure in the setting of severe preeclampsia, so an emergent left heart catheterization was performed, which revealed normal coronary arteries (Figure 2). 

**Table 1:** Cardiac profile results.

Troponin I (reference range <16 ng/L)	
0 hour	23,949 ng/L
2 hour	69,890 ng/L
6 hour	20,953 ng/L
BNP (reference range: 10.0–100.0 pg/mL)	45.2 pg/mL

**Figure 3:**
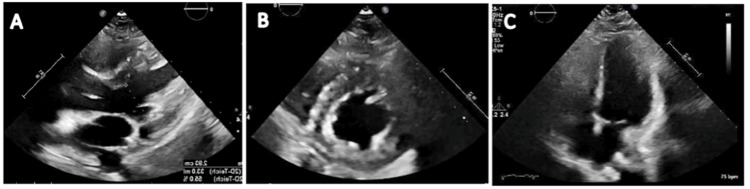
Transthoracic echocardiogram images showing parasternal long axis (A), parasternal short axis (B), and apical four chamber views (C). There is normal ejection fraction, normal left ventricular wall thickness, normal wall motion, systolic and diastolic functions.

**Figure 4:**
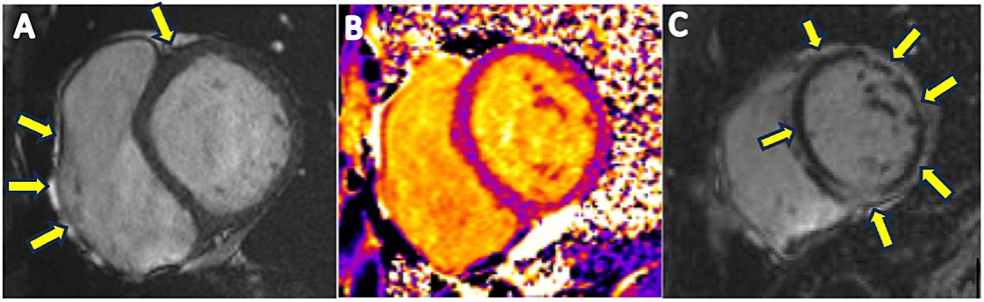
Short axes images of cardiovascular magnetic resonance images showing abnormal delayed enhancement (arrows).

The transthoracic echocardiogram (TTE) showed an ejection fraction of 50-55%, normal left ventricular wall thickness, normal wall motion, and normal systolic and diastolic functions (Figure 3). There were no structural abnormalities. The erythrocyte sedimentation rate was elevated at 34 mm/HR (reference range: 0-20 mm/HR). The patient was started on treatment for myopericarditis with aspirin and colchicine. Cardiac magnetic resonance imaging was done, which revealed normal myocardial wall thickness and chamber dimensions with severely delayed enhancement suggestive of myocarditis (Figure 4).

**Figure 5:**
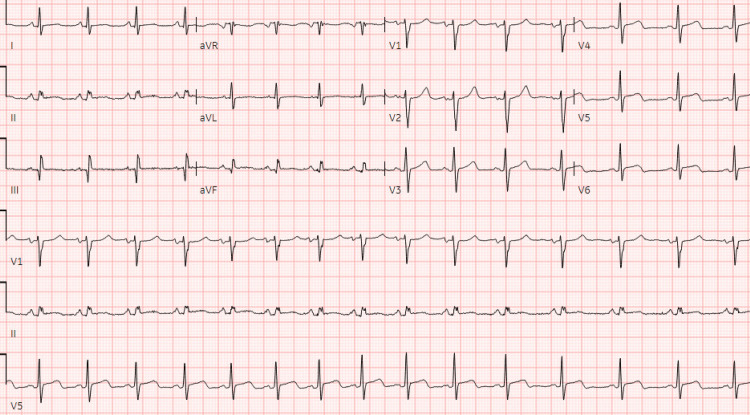
Normal sinus rhythm with sinus arrhythmia and lateral ST elevation.

An extensive workup for the infectious etiology of myocarditis revealed positive Coxsackie B5 antibody and Epstein Bar virus immunoglobulin G titers were elevated at 1:8 (reference range <1:8) and 191 (reference range <18 U/mL), respectively, which often signify previous infection. The antinuclear antibody (ANA) screen was positive. No other specific markers were reactive, including parvovirus B19 antibody, hepatitis A, B, and C antibodies, cytomegalovirus DNA, COVID-19, influenza A and B, RSV, legionella antigen, enterovirus RNA, adenovirus DNA, and HIV. The patient was discharged the next day to complete myocarditis treatment with colchicine and taper a dose of aspirin. Nifedipine was substituted for metoprolol as part of guideline-directed management for heart failure with reduced ejection fraction, with plans to follow up with our heart failure clinic. Angiotensin-converting enzyme inhibitor was not initiated as the patient had low blood pressure (blood pressure on admission was 129/98 mmHg and 109/70 mmHg on discharge). The discharge EKG showed normal sinus rhythm with sinus arrhythmia and lateral ST elevation (Figure 5). Repeat troponins one month following discharge revealed normal troponins. Follow-up CMRI nine months after discharge showed an improved left ventricular ejection fraction (LVEF) of 55% but persistently delayed gadolinium enhancement. Table 2 summarizes key events.

**Table 2:** Timeline of significant events.

Time	Events
5 years prior to presentation	Admitted and treated for myocarditis, confirmed by MRI. Normal coronary CTA.
12 days prior to presentation	Preterm delivery by Caesarean section on account of severe pre-eclampsia and HELLP syndrome.
Day 0 (day of presentation)	Chest pain, elevated troponin, anterior-lateral ST elevation, and accelerated junctional rhythm on EKG.
	Emergent left-heart catheterization showed normal coronary arteries.
	TTE: LVEF 50–55% with normal LV wall motion.
	Aspirin and colchicine started.
Day 1	Cardiac MRI shows reduced LVEF of 37% with extensive findings of hyperenhancement consistent with myocarditis.
	Normal sinus rhythm with sinus arrhythmia and lateral ST elevation. Patient is discharged.
Day 32	Repeat TTE and troponin show normalized troponin level and LVEF of 55–60%.
9 month	Cardiac MRI shows persistent delayed gadolinium enhancement and improved EF of 55%.

## Discussion

While there are various infectious and non-infectious etiologies of acute myocarditis, viral etiologies are the most common causes of the disease [5]. Prior to the COVID-19 pandemic, common viral causes of acute myocarditis included coxsackievirus, adenovirus, cytomegalovirus, and parvovirus B19, as well as hepatitis C, influenza, herpes simplex, and Epstein-Barr viruses [6]. Currently, the COVID-19 disease and its vaccines seem to be contributing to an increased incidence of acute myocarditis [2]. The disease has also been associated with autoimmune disorders such as sarcoidosis, giant cell arteritis, systemic lupus erythematosus, and ulcerative colitis [1,5,7]. Furthermore, drugs and vaccines such as checkpoint inhibitors, smallpox, and COVID mRNA vaccines have been identified as known causes of myocarditis [5]. In this patient presentation, Coxsackievirus B5 and Epstein-Barr virus antibodies were positive. The positive Epstein-Barr test was an IgG test, which signifies a previous infection and cannot account for the patient's current myocarditis; the IgM test, which is positive for acute infection, was negative in this patient. The patient's coxsackievirus could mean either an acute or a past infection; a fourfold titer increase in the convalescent period compared to the acute period would have confirmed an acute infection [8]. However, we did not do this for our patient because it would not have influenced management.

Up to 95% of patients experience chest pain, the most common presentation of acute myocarditis. Other symptoms of the disease include fever, dyspnea, and syncope. Acute myocarditis is often preceded by a prodrome of flu-like symptoms, gastrointestinal disorders, or upper respiratory tract infections, days to weeks before the disease. The disease course is varied and often uncomplicated, with rapid recovery, but it could also lead to acute heart failure, cardiogenic shock, dilated cardiomyopathy, and life-threatening arrhythmias and conduction disturbances. In severe cases where there is hemodynamic instability and the requirement for ionotropic agents, the mortality rate could be as high as 28%, with an increased probability of needing a cardiac transplant [5,9]. In this case report, the patient experienced a rapid worsening of the disease, with LVEF worsening from 50% to 55% to 37% within 24 hours of presentation; however, she remained hemodynamically stable. Her LVEF improved relatively rapidly, with repeat TTE showing LVEF of 55% to 60% a month after presentation.

Diagnostic investigations, besides infectious workup, may include nonspecific tests such as EKG, TTE, and cardiac troponins. Myocarditis can present a variety of EKG findings, from normal EKG, which may be present in up to 15% of patients, to injury pattern ST-segment changes. Conduction abnormalities may also be observed. EKG findings associated with worse outcomes include QRS prolongation above 120 ms or the presence of Q-waves [1,6,10]. As was the case with our patient, troponins are commonly elevated. An echocardiogram may be useful in the initial triaging of patients, given the correlation between reduced LVEF and adverse outcomes [10]. The finding of late gadolinium enhancement on cardiac magnetic resonance imaging is a common way to confirm the diagnosis of acute myocarditis, as seen in our patient. Additionally, complete MRI findings for our patient included epicardial enhancement involving almost the entire left ventricle, only sparing the distal anterior wall. For a selected category of patients, however, immunohistology evidence with an endomyocardial biopsy (EMB) is required for diagnosis. Such patients include those with cardiogenic shock, new-onset ventricular arrhythmias, peripheral eosinophilia or an associated systemic inflammatory disorder, persistently elevated troponins, or cardiac dysfunction in a patient receiving immune checkpoint inhibitor therapy [9,10].

Among patients diagnosed with recurrent myocarditis, late recurrence (>1 year after initial diagnosis) is rare. Indeed, a nationwide retrospective cross-sectional study using the Finnish Hospital Discharge Register (FHDR) revealed a late recurrence of 4.7% with a median time to late-onset recurrence of 2.3 years [3]. While the pathogenesis of recurrence is not fully understood, a number of mechanisms have been suggested, including the development of autoantibodies and autoreactive T-cells against various components of cardiac myocytes [11-13]. The process of autoantibody generation is part of the initial body reaction in viral myocarditis and may cause a recurrence of cardiac inflammation following the resolution of the initial viral insult [1,11]. Another postulated mechanism is the reactivation of a latent viral infection. Several viruses known to cause myocarditis, such as adenovirus, enterovirus/coxsackievirus, parvovirus B19, herpes simplex virus, and cytomegalovirus infection, may persist in a latent form or as chronic infections following the initial infection [14]. It is also reasonably possible that one gets repeated exposure to a causative organism, leading to the recurrence of myocarditis.

PPCM is a differential diagnosis to be considered in our patient, given the fact that she presented in the peripartum period with pedal edema, an LVEF of 37%, and the risk factor of preeclampsia. However, PPCM is a diagnosis of exclusion [4]. Our patient had findings more in keeping with the diagnosis of acute myocarditis. Treatment of myocarditis involves treating the underlying cause and associated complications. Immunosuppressive therapy is indicated for specific myocarditis types, such as eosinophilic myocarditis and giant cell myocarditis. Our patient received aspirin and colchicine because she also had involvement in her pericardium.

## Conclusions

This case report provides a rare case of a late recurrence of myocarditis in a postpartum patient. The diligent work done to arrive at the right diagnosis instead of anchoring on PPCM is of important educational value. While the clinical course of the disease is often benign, it could rapidly deteriorate, so early recognition and diagnosis are important. Importantly, patients with a history of myocarditis ought to be educated about the recurrence risk so that they can seek early medical care.
